# Post-mastectomy pain syndrome as a model for mixed pain: clinical evidence from a specialized cancer pain clinic

**DOI:** 10.3389/fmed.2026.1733623

**Published:** 2026-04-15

**Authors:** Morsi Khashan, Silviu Brill, Yehonatan Hochberg, Uri Hochberg

**Affiliations:** 1 Gray Faculty of Medical & Health Sciences, Tel Aviv University, Tel Aviv, Israel; 2 Tel Aviv Sourasky Medical Center, Tel Aviv, Israel

**Keywords:** breast cancer surgery, catastrophizing, mechanism-based therapy, mixed pain, neuropathic pain, nociceptive pain, post-mastectomy pain syndrome

## Abstract

**Introduction:**

Post-mastectomy pain syndrome (PMPS) is a common and disabling complication after breast cancer surgery. Although traditionally categorized as neuropathic or nociceptive, many patients present with overlapping features consistent with mixed pain, a phenotype that remains poorly defined in clinical practice.

**Methods:**

We performed a retrospective analysis of prospectively maintained data from women with refractory PMPS referred to a tertiary cancer-related pain clinic between January 2022 and September 2025. Pain was classified as nociceptive, neuropathic, or mixed based on structured clinical assessment. Patient-reported measures included the Brief Pain Inventory (BPI) and Pain Catastrophizing Scale (PCS). Multivariable logistic regression was used to examine factors independently associated with mixed pain.

**Results:**

One hundred twenty women (mean age 57.9 ± 12.5 years) were included. Pain phenotypes were nociceptive in 40.8%, neuropathic in 25.0%, and mixed in 34.2%. Pain intensity, interference, and catastrophizing scores were elevated across groups without statistically significant differences (all *p* > 0.05). In adjusted analyses (analytic *N* = 113), multiplicity of pain sources (≥2 concurrent pain generators; adjusted OR 49.96; 95% CI 11.69–213.41) and radiotherapy-attributed pain (adjusted OR 5.75; 95% CI 1.15–28.82) were independently associated with mixed pain. Model stability was evaluated in sensitivity analyses using Firth’s penalized likelihood logistic regression.

**Conclusion:**

In this tertiary cohort of women with refractory PMPS, mixed pain accounted for approximately one-third of cases and was independently associated with multiple concurrent pain sources and radiotherapy-attributed pain. These findings suggest that the coexistence of multiple pain sources, rather than pain severity alone, may characterize mixed pain presentations. Given the observational design and model limitations, results should be interpreted as hypothesis-generating. Mechanism-based assessment may help inform individualized management strategies.

## Introduction

Breast cancer is the most common malignancy among women worldwide, and advances in surgery, systemic therapy, and radiotherapy have significantly improved survival outcomes (1). Surgical procedures, including mastectomy and breast-conserving surgery, remain essential for disease control. However, improved survival has been accompanied by an increasing recognition of long-term treatment-related morbidity, particularly persistent postsurgical pain syndromes that adversely affect quality of life, physical functioning, and psychosocial well-being (2). Post-mastectomy pain syndrome (PMPS) is among the most prevalent of these complications, affecting approximately 25–60% of women after breast cancer surgery (2).

Despite its high prevalence, PMPS remains a poorly defined and heterogeneous entity, with no consensus on standardized diagnostic criteria (3, 4). Definitions vary across studies in terms of anatomical boundaries, required symptom duration, and mechanistic interpretation (3–6). Although PMPS was introduced in the late 1970s and initially conceptualized as predominantly neuropathic pain attributed to intercostobrachial nerve injury (7, 8), it is now understood as a multifactorial condition involving neuropathic, nociceptive, musculoskeletal, and radiation-related components rather than a single causal pathway (3, 5, 9, 10). This definitional variability contributes to inconsistent epidemiological estimates and complicates clinical recognition and mechanism-based treatment planning (3, 4, 11).

Contemporary pain frameworks increasingly recognize that chronic postsurgical pain often reflects overlapping peripheral and central mechanisms rather than a single mechanistic category. The ICD-11 classification of chronic pain and contemporary neuropathic pain frameworks acknowledge the frequent overlap between nociceptive, neuropathic, and nociplastic mechanisms (11–13). In this context, the concept of “mixed pain” has emerged to describe clinical presentations in which nociceptive and neuropathic features coexist within the same anatomical region (14, 15). Although mixed pain has been discussed in cancer-related and non-cancer pain conditions, consensus diagnostic criteria remain underdeveloped, and empirical characterization in specific clinical syndromes is limited (14, 16).

Given the frequent coexistence of surgical nerve injury and musculoskeletal tissue disruption, PMPS may serve as a clinically relevant model of mixed pain. Surgical nerve injury, musculoskeletal trauma related to axillary dissection or reconstruction, radiotherapy-associated fibrosis, and central sensitization may interact to generate overlapping nociceptive and neuropathic mechanisms (4, 13, 16, 17). Although PMPS is increasingly recognized as mechanistically heterogeneous, systematic data quantifying mixed pain presentations—particularly in tertiary, treatment-refractory populations—remain limited.

Over the past decade, our institution has operated a dedicated Cancer-Related Pain Clinic that integrates interventional and surgical expertise to address refractory cancer-related pain. Patients are referred after exhausting guideline-based pharmacologic therapy according to the World Health Organization (WHO) analgesic ladder (18, 19), resulting in a cohort with complex and treatment-resistant pain.

In this study, we sought to characterize the distribution of nociceptive, neuropathic, and mixed pain type among women with refractory PMPS referred to a specialized cancer pain clinic and to examine clinical factors independently associated with the mixed pain presentation. Rather than presuming PMPS to be predominantly neuropathic, we aimed to provide empirical data to examine its underlying mechanisms and assess whether the presence of multiple pain sources is associated with a mixed pain presentation.

## Methods

### Study design and oversight

This is a retrospective analysis of prospectively collected data from the Cancer-Related Pain Clinic of a tertiary, university-affiliated medical center. The institutional ethics committee approved the study (TLV 0219–23).

The clinic’s data were collected prospectively as part of routine standardized care; however, the specific hypotheses and statistical analyses for the present study were defined after completion of data collection.

### Patients

The study included consecutive eligible women with refractory pain who were referred to the Cancer-Related Pain Clinic for consideration of interventional procedures between January 2022 and September 2025. Eligible patients had undergone mastectomy or breast-conserving surgery for breast cancer and reported persistent pain in the chest wall, axilla, or ipsilateral upper limb. All referrals originated from the hospital’s oncology and palliative care services following unsuccessful guideline-based pharmacologic therapy according to the World Health Organization (WHO) analgesic ladder (18, 19), including escalation through opioid and adjuvant analgesic treatments when clinically appropriate. Accordingly, this cohort represents a highly selected tertiary-level population rather than a general PMPS cohort typically included in epidemiological studies.

Prior to clinical evaluation, a formal triage process confirmed refractory pain status and suitability for interventional pain management. Patients were assessed by a pain specialist in a multidisciplinary setting offering surgical and image-guided procedures, including joint injections, peripheral nerve and fascial plane blocks, epidural steroid injections, and neuromodulation techniques.

### Pain diagnosis and classification

Pain diagnosis was based on a structured clinical evaluation performed by trained pain specialists, integrating patient history, physical examination, and neurologic assessment. Diagnostic assessment included evaluation of sensory descriptors, pain distribution, presence of allodynia or dysesthesia, musculoskeletal dysfunction, and functional impairment. Pain was classified as nociceptive, neuropathic, or mixed.

Neuropathic pain was classified in accordance with the NeuPSIG 2016 grading system (12). Classification required a neuroanatomically plausible pain distribution and a relevant lesion or disease affecting the somatosensory system (e.g., surgery- or radiotherapy-related nerve injury), together with confirmatory clinical evidence of somatosensory system involvement. Confirmatory evidence included neuropathic sensory descriptors and/or examination findings (e.g., allodynia, hyperalgesia, or sensory loss) and, in the majority of cases, objective confirmation through one or more of the following when available as part of routine clinical care: electrophysiologic studies (EMG/nerve conduction), imaging demonstrating nerve root or plexus involvement, surgical documentation of nerve injury, radiologically confirmed radiation-induced plexopathy, or sensory loss in a neuroanatomically consistent distribution.

Nociceptive pain referred to musculoskeletal or soft-tissue pain without neuropathic descriptors, and mixed pain was assigned when both nociceptive and neuropathic features were present in the same site. Pain sources were documented anatomically and by mechanism, and patients were categorized as having one versus ≥2 concurrent pain generators.

All patients were evaluated using a standardized intake protocol routinely implemented in the Cancer-Related Pain Clinic. This protocol includes a structured pain history template, systematic neurologic examination, predefined documentation of neuropathic features consistent with NeuPSIG criteria, and formal recording of pain type (nociceptive, neuropathic, or mixed) in a designated field within the electronic medical record. Pain classification was assigned prospectively at the time of clinical evaluation rather than retrospectively reconstructed for research purposes.

Evaluations were performed by board-certified pain specialists trained in the clinic’s standardized assessment framework. In cases of diagnostic uncertainty or complex presentations, pain type were discussed among clinic staff and, when appropriate, with palliative care physicians to achieve consensus classification. Formal inter-rater reliability testing was not conducted.

### Criteria

Inclusion criteria were: (1) female sex, aged ≥18 years; (2) breast cancer surgery (mastectomy or breast-conserving surgery); (3) persistent chest wall, axillary, or ipsilateral upper limb pain attributed to breast surgery; (4) referral to the cancer-related pain clinic after inadequate response to WHO analgesic ladder–based therapy.

Exclusion criteria were surgery for benign or non-oncological conditions, or pain deemed unrelated to breast surgery by the evaluation specialist.

### Pain assessments

Patient-reported outcome measures included the Brief Pain Inventory (BPI) for pain severity and interference (20) and the Pain Catastrophizing Scale (PCS) to assess pain-related catastrophizing (21). PCS total scores ranged from 0 to 52 and were calculated as the sum of the 13 items. Scores ≥30 were considered indicative of clinically elevated catastrophizing, consistent with published PCS guidelines (22).

Completion of patient-reported outcome measures occurred as part of the routine clinical workflow; missing data primarily reflected incomplete questionnaires or time constraints during clinic visits, and did not differ by pain type. There was no systematic clinical exclusion from the administration of questionnaires.

Clinical variables included age, type of surgery, time since surgery, radiotherapy exposure, systemic and hormonal therapies, the presence of lymphedema, fibromyalgia, peripheral neuropathy, and referral to interventional pain procedures.

### Multivariable analysis of factors independently associated with mixed pain

To examine factors independently associated with mixed pain (vs nociceptive or neuropathic pain), we fitted a multivariable logistic regression model with mixed pain as the dependent variable (mixed = 1; nociceptive/neuropathic = 0). Prespecified demographic, surgical, and treatment-related clinical variables were included: age, type of surgery (plain vs. reconstruction), time-from-surgery, current systemic treatment, current hormonal treatment, radiotherapy-attributed pain, multiplicity of pain sources (≥2 vs. single), lymphedema, later diagnosis of fibromyalgia, peripheral neuropathy, and interventional block variables (block suggested and block carried).

Patient-reported outcome measures (Pain Catastrophizing Scale and pain interference score) were analyzed descriptively and were not included in the primary multivariable model to avoid reduction in analytic sample size due to missing data.

The regression model was fit using complete-case covariate data. All 120 patients had available pain type classification; seven patients were excluded due to missing/invalid covariate coding (time-from-surgery missing/invalid in 4 cases and fibromyalgia coded non-binary in 3 cases), resulting in an analytic sample of *N* = 113. All prespecified variables were retained without stepwise selection.

### Statistical analysis

Continuous variables are presented as means with standard deviations or medians with interquartile ranges, and categorical variables as counts and percentages. Between-group comparisons used one-way ANOVA or Kruskal–Wallis tests for continuous variables, as appropriate, and chi-square tests for categorical variables. When overall tests were significant, Tukey’s method was applied for *post-hoc* comparisons.

Univariate analyses were used to describe the strength and direction of associations. Results from logistic regression are reported as odds ratios (ORs) for univariate models and adjusted odds ratios (aORs) with 95% confidence intervals. Missingness of patient-reported outcomes across pain type was assessed using chi-square tests. All tests were two-sided, with a significance threshold of *p* < 0.05. Analyses were performed using available case data for descriptive comparisons and complete-case covariate data for regression models, without imputation. All reported statistical results were independently verified against the original dataset.

To evaluate potential sparse-data bias and mitigate (quasi-) separation, we conducted a sensitivity analysis using Firth’s penalized likelihood logistic regression, modeling mixed pain (mixed vs. nociceptive/neuropathic) and including the same prespecified covariates as in the primary multivariable model.

## Results

A total of 120 patients were included (mean [SD] age, 57.9 [12.5] years). Most underwent plain surgery (74.2%) and the remainder reconstructive surgery (25.8%). Over half of the cohort (55.0%) was beyond 1 year from surgery, and lymphedema was present in 34.2%.

The mean (SD) pain score was 25.8 ± 7.8 and the pain interference score was 45.0 ± 16.4. The mean (SD) PCS score was 31.1 ± 13.4 (Table 1). Overall, approximately three-fourths of participants had available pain and interference data (75–78%), and two-thirds completed the PCS assessment (67.5%).

**Table 1:** Clinical and surgical characteristics of the study population.

Characteristic	Mean ± SD	Number (%)
Demographic		
Age, years	57.9 ± 12.5	120 (100%)
Pain measures		
Pain items score, total	25.8 ± 7.8	90 (75.0%)
Pain interference items score, total	45.0 ± 16.4	93 (77.5%)
Pain catastrophizing scale (PCS), total	31.1 ± 13.4	81 (67.5%)
Pain type		
Nociceptive		49 (40.8%)
Neuropathic		30 (25.0%)
Mixed		41 (34.2%)
Surgical characteristics		
Type of surgery—plain		89 (74.2%)
Type of surgery—with reconstructive surgery		31 (25.8%)
Time from surgery—< 6 months		18 (15.0%)
Time from surgery—6–12 months		32 (26.7%)
Time from surgery— > 12 months–<5 years		33 (27.5%)
Time from surgery— > 5 years		33 (27.5%)
Lymphedema present		41 (34.2%)

Values are presented as mean ± standard deviation (SD) or n (%). Percentages are calculated from the total cohort (*N* = 120). Time-from-surgery data were available for 116/120 patients (96.7%); 4 patients had missing/invalid values. PCS, Pain Catastrophizing Scale.

Pain types were distributed as nociceptive 40.8%, neuropathic 25.0%, and mixed 34.2%. The anatomical distribution of pain sources is summarized in Table 2. The most common region was the chest wall/thoracic rib cage (31.7%), followed by shoulder pain (9.2%) and diffuse upper-quadrant pain (7.5%). Notably, 34 patients (28.3%) exhibited ≥2 concurrent pain generator. In sensitivity analyses using Firth’s penalized likelihood logistic regression, the association between multiplicity of pain sources and mixed pain remained robust (Firth aOR 24.58; 95% CI 7.11–84.95; *p* < 0.001). The association between radiotherapy-attributed pain and mixed pain was attenuated and did not reach statistical significance (Firth aOR 3.53; 95% CI 0.86–14.48; *p* = 0.080). Full penalized regression results are provided in Supplementary Table 6.

**Table 2:** Univariate comparison of predictors by pain group.

Variable	Type	*p* value	Significant
Pain source	Categorical	**0.0000**	*
Pain due to radiation	Categorical	**0.0031**	*
Time from surgery	Categorical	0.3205	
PCS score	Continuous	0.3333	
Age	Continuous	0.3368	
Later diagnosis of fibromyalgia	Categorical	0.5771	
Pain intervention score	Continuous	0.5950	
Lymphedema	Categorical	0.8418	
Systemic treatment	Categorical	0.8701	
An interventional procedure was suggested	Categorical	0.9057	
An interventional procedure was carried	Categorical	0.9057	
Type of surgery	Categorical	0.9370	
Hormonal treatment	Categorical	0.9637	
Peripheral neuropathy	Categorical	1.0000	

Univariate analyses comparing patients with mixed pain (*n* = 35) to those with nociceptive or neuropathic pain (*n* = 85). Categorical variables analyzed using χ^2^ tests; continuous variables using Kruskal–Wallis tests. Significant values are bolded (*p < 0.05). PCS, Pain Catastrophizing Scale. Significant differences determined at *p* < 0.05. Primary pain source categories reflect the predominant anatomical and mechanistic pain generator identified during structured clinical assessment. Diffuse upper-quadrant pain and diffuse regional pain represent distinct single-mechanism entities (e.g., radicular/plexopathy or myofascial pain) and should not be interpreted as equivalent to “multiple pain sources,” which reflects the presence of ≥2 concurrent pain mechanisms identified in the same or different anatomical regions. Percentages are calculated from the total cohort (*N* = 120).

The full cohort included 41 patients classified as having mixed pain; however, because the multivariable regression model used complete-case covariate data (analytic *N* = 113), 3 mixed-pain cases were excluded due to missing or invalid covariate data, leaving 38 mixed-pain cases in the analytic sample.

### Pain and PCS scores by pain type

The mean (SD) pain score was 25.4 ± 6.9 (median 26.0) in the nociceptive group, 26.8 ± 10.2 (median 26.5) in the neuropathic group, and 26.8 ± 6.9 (median 27.0) in the mixed-pain group (overall *p* = 0.666).

For the Pain Catastrophizing Scale (PCS), among patients with available PCS data (*n* = 81), nociceptive patients scored 29.3 ± 13.0 (median 31.0; *n* = 34), compared with 31.5 ± 15.5 (median 33.5; *n* = 20) in the neuropathic group and 33.2 ± 12.4 (median 37.0; *n* = 27) in the mixed-pain group (overall *p* = 0.075). Data availability did not differ significantly across pain type for pain score, pain interference, or PCS (all χ^2^ tests *p* > 0.55). Using a threshold of PCS ≥ 30 to indicate clinically elevated catastrophizing, 18/34 (52.9%), 12/20 (60.0%), and 20/27 (74.1%) patients met this criterion in the nociceptive, neuropathic, and mixed groups, respectively.

**Figure 1:**
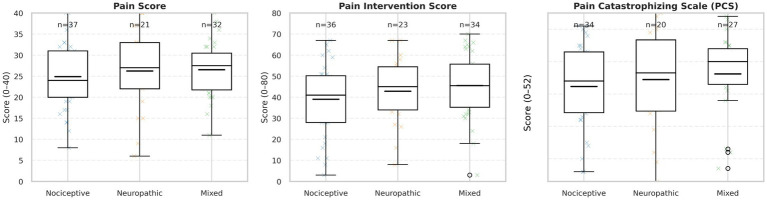
Comparison of pain measures by pain type. Boxplots display Pain (0–40), Pain Interference (0–80), and Pain Catastrophizing Scale (PCS; 0–52) scores across nociceptive, neuropathic, and mixed pain groups. Mixed-pain patients showed numerically higher values in all domains, though differences were not statistically significant (all *P* > .05). Boxes indicate interquartile ranges (IQRs); lines show medians; whiskers extend to 1.5 × IQR; points represent individual participants; and short horizontal ticks mark group means. “n =” indicates group sample size. Sample sizes differ across panels due to missing patient-reported outcome data.

Although mean values were numerically higher in the mixed-pain group, between-group differences did not reach statistical significance (Figure 1).

### Predictors of mixed pain

**Figure 2:**
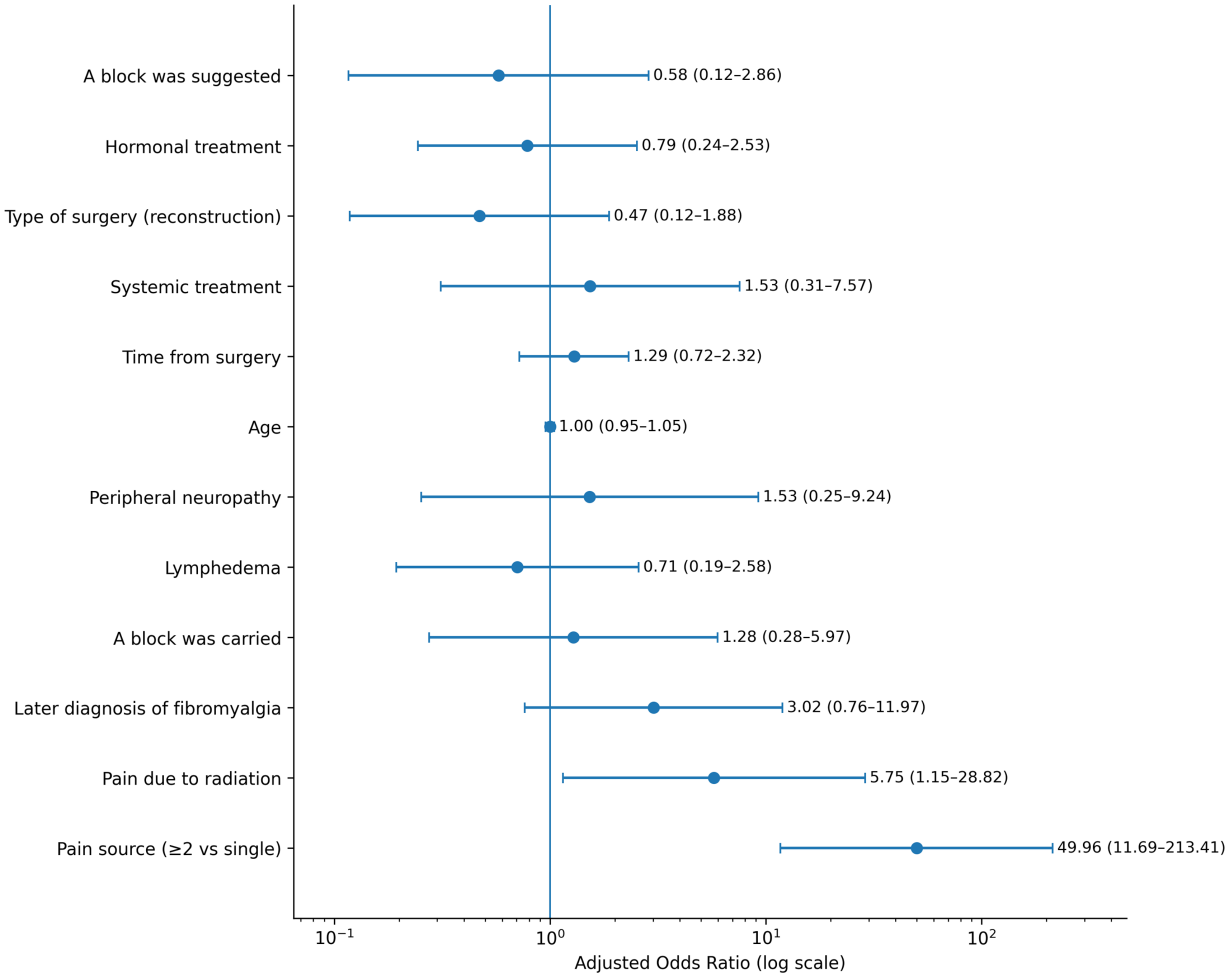
Multivariable factors independently associated with mixed pain. Forest plot displaying adjusted odds ratios (aORs) with 95% confidence intervals (CIs) from a multivariable logistic regression model comparing mixed pain versus nociceptive or neuropathic pain (analytic N = 113). The solid vertical line indicates aOR = 1 (no association). Variables independently associated with mixed pain included multiplicity of pain sources (≥2 concurrent pain generators) and pain attributed to radiotherapy.

In multivariable logistic regression including demographic, surgical, and treatment-related covariates (analytic *N* = 113), multiplicity of pain sources (*β* = 3.91; *p* < 0.001; adjusted aOR 49.96; 95% CI 11.69–213.41) and radiotherapy-attributed pain (*β* = 1.75; *p* = 0.033; adjusted aOR 5.75; 95% CI 1.15–28.82) were independently associated with mixed pain. No other covariates demonstrated statistically significant associations in the adjusted model (Figure 2). Full model results are presented in Supplementary Tables 3, 4.

The cross-tabulation of pain source multiplicity (≥2 vs. single) and mixed pain classification in the analytic sample (*N* = 113) is shown in Supplementary Table 5; no zero cells were observed.

### Interventional pain procedures

Interventional procedures were suggested in 72 patients (60.0%). Among 41 patients classified as having mixed pain, recommendation data were available for 40; of these, 25 (62.5%) were advised to undergo an interventional procedure. Corresponding rates were 15 of 30 patients (50.0%) in the neuropathic group and 32 of 49 patients (65.3%) in the nociceptive group. Differences between pain type were not statistically significant (*p* = 0.38).

Procedures were ultimately carried out in 47 patients (39.2%). Among 41 patients classified as having mixed pain, procedure data were available for 40; of these, 15 (37.5%) underwent blocks. Corresponding rates were 9 of 30 (30.0%) in the neuropathic group and 23 of 49 (46.9%) in the nociceptive group. There were no statistically significant differences between pain type (*p* = 0.31).

Among the 72 patients for whom procedures were recommended, 25 did not undergo intervention during the study observation period. This reflected patient preference (declining or deferring intervention), interim clinical improvement, medical contraindications identified during assessment, or incomplete follow-up, consistent with routine tertiary-care clinical practice.

## Discussion

In this cohort of women with refractory post-mastectomy pain syndrome (PMPS), mixed pain presentations were common and demonstrated clinical features that differed from cases classified as predominantly nociceptive or neuropathic. The coexistence of multiple pain sources showed a strong independent association with mixed pain classification, suggesting that mechanistic complexity may be clinically relevant in the assessment of persistent PMPS. To our knowledge, few studies have systematically evaluated factors associated with mixed pain presentations in PMPS, and the present findings contribute empirical data supporting an association between multiplicity of pain sources and mixed pain classification. Although PMPS has traditionally been conceptualized as predominantly neuropathic—often attributed to intercostobrachial nerve injury—the distribution of pain type in this cohort suggests that nerve injury alone may not sufficiently explain the clinical heterogeneity observed in refractory cases. Instead, the findings are consistent with converging contributions from musculoskeletal factors, radiotherapy-related tissue changes, neuropathic mechanisms, and processes compatible with central sensitization. Together, these observations support the interpretation that mixed pain in PMPS may reflect a multidimensional clinical pattern rather than a single dominant mechanism.

The anatomical distribution of pain sources in this cohort (Table 2) further illustrates the clinical complexity of PMPS. The chest wall and thoracic rib cage constituted the most common region of pain (31.7%), reflecting the combined impact of surgical trauma, radiotherapy-related fibrosis, and neuropathic injury. The frequent coexistence of multiplicity of pain sources (28.3% with ≥2 sources) indicates that PMPS is rarely attributable to a single mechanism such as isolated intercostobrachial nerve injury and supports the need for mechanism-based assessment and treatment strategies.

The most robust independent association with mixed pain classification was the presence of multiple pain sources. A substantial proportion of patients classified as having mixed pain also reported multiple concurrent pain generators. However, multiplicity of pain sources reflects the presence of ≥2 clinically identified pain generators and does not, by definition, require the coexistence of nociceptive and neuropathic mechanisms within the same anatomical site. As such, multiplicity and mixed pain classification represent related but distinct constructs.

To further evaluate the potential impact of small-sample bias and near-separation, we performed a sensitivity analysis using Firth’s penalized likelihood logistic regression including the same prespecified covariates. In this model, multiplicity of pain sources remained independently associated with mixed pain (Firth aOR 24.58; 95% CI 7.11–84.95; *p* < 0.001), whereas the association between radiotherapy-attributed pain and mixed pain was attenuated and did not reach statistical significance (Firth aOR 3.53; 95% CI 0.86–14.48; *p* = 0.080). Together, these findings suggest that the association between multiplicity of pain sources and mixed pain is consistent under penalized estimation, whereas the radiotherapy-attributed pain association appears more sensitive to model specification.

The large adjusted odds ratio observed for multiplicity of pain sources should be interpreted cautiously. This tertiary, treatment-refractory cohort likely enriched for clinically complex presentations, limiting generalizability and potentially inflating effect estimates. The number of mixed-pain events in the analytic sample was modest relative to the number of prespecified covariates, yielding an events-per-variable ratio below conventional thresholds and increasing vulnerability to overfitting and sparse-data bias (including quasi-separation and coefficient inflation), as reflected in the wide confidence interval. Although the Firth sensitivity analysis supports the direction of association, uncertainty remains regarding the precise magnitude of effect. Because multiplicity and mixed pain are related but not identical constructs, the strength of association may also partly reflect their conceptual proximity.

Radiotherapy was also independently associated with mixed pain classification in this study. This association may reflect late tissue effects, including progressive cutaneous and subcutaneous fibrosis that may persist or worsen years after treatment (23–25). Given the observational design, this finding should be interpreted as an association rather than evidence of a causal mechanistic effect.

These observations have important clinical implications. If multiple pain sources—rather than pain severity alone—characterize the mixed pain presentation, escalation of therapy based solely on severity may be insufficient. Instead, a mechanism-based approach is warranted: clinicians should identify musculoskeletal pain drivers, neuropathic components, and radiotherapy-related contributors, and address each specifically.

Potential interventions (e.g., regional nerve blocks or targeted soft-tissue procedures) may be conceptually aligned with a mechanism-based framework; however, because treatment approaches and outcomes were not assessed in the present study, this discussion is offered as clinical context and should not be interpreted as a conclusion supported by the present data. In patients with a history of radiotherapy, fibrosis and impaired tissue healing pose additional challenges, as even minimally invasive interventions—such as nerve blocks or soft-tissue injections—may carry an increased risk of ulceration or infection (23–25). In many cases, multimodal strategies remain necessary due to the coexistence of multiple pain mechanisms, encompassing pharmacologic, interventional, rehabilitative, and psychosocial modalities (8, 10).

Across the entire cohort and within each pain-type subgroup, participants demonstrated elevated levels of pain catastrophizing. The mean PCS score exceeded the commonly used clinical threshold of 30, suggesting clinically elevated levels of catastrophizing in this cohort (22). Although between-group differences were not statistically significant, numerically higher PCS scores in the mixed pain group are consistent with prior literature linking overlapping nociceptive and neuropathic mechanisms to enhanced pain processing and increased clinical complexity (13, 26). The mixed pain presentation may reflect the convergence of peripheral and central sensitization processes, which have been associated with amplified pain perception and reduced treatment responsiveness (13). In breast cancer patients, higher levels of pain catastrophizing have been correlated with greater self-reported features of central sensitization, supporting an interaction between psychological and biological sensitization mechanisms (4, 26).

Pain catastrophizing is associated with heightened pain perception, hypervigilance, and persistent pain in chronic pain populations (21, 26). Accordingly, incorporating psychological assessment and intervention into multimodal pain management may be beneficial. Collectively, these findings are consistent with the hypothesis that mixed pain in PMPS may represent a multidimensional clinical pattern characterized by overlapping peripheral and central mechanisms; however, in the absence of standardized diagnostic criteria, this interpretation remains provisional.

Accurate diagnosis of mixed pain remains clinically challenging. Tools such as painDETECT, a patient-reported screening questionnaire designed to distinguish nociceptive from neuropathic mechanisms (27), may misclassify mixed pain and underestimate its prevalence. This limitation highlights the urgent need for validated tools that can detect overlapping mechanisms and more precisely characterize mixed pain (5, 14, 17).

Although the present analysis was limited to refractory PMPS in a tertiary-care setting, these findings may have broader implications beyond PMPS. By demonstrating that multiplicity of pain sources is independently associated with mixed pain classification, our findings are consistent with the rationale for integrating nociceptive, neuropathic, and nociplastic mechanisms (9, 11, 14, 15). Establishing standardized definitions may improve clinical classification, patient outcomes, refine clinical trial enrollment, and accelerate the development of specific multimodal therapies. Recent regional initiatives, such as the Latin American statement on mixed pain, highlight both urgency and the feasibility of creating a common framework across diverse health systems (16).

This study has several limitations. As the study was retrospective and conducted at a single tertiary-care center addressing a highly selected population, its findings may not be broadly generalizable. The retrospective design also precludes temporal or causal inference. The sample size within each pain subgroup was relatively small, restricting analyses. Given the number of mixed pain cases (38 mixed-pain events in the analytic regression sample, N = 113) relative to the number of covariates included in the multivariable model, the events-per-variable ratio was below conventional recommendations; therefore, effect size estimates should be interpreted cautiously and considered exploratory.

Although a sensitivity analysis using Firth’s penalized likelihood logistic regression was performed to mitigate potential sparse-data bias and quasi-separation, residual small-sample instability cannot be entirely excluded. In addition, the magnitude of the association between multiplicity of pain sources and mixed pain should be interpreted in the context of conceptual proximity between these constructs.

Longitudinal outcome data were not available to evaluate changes in pain characteristics over time. Pain classification was based on expert clinical judgment, and while neuropathic pain was classified according to NeuPSIG criteria, no validated diagnostic criteria or standardized tools currently exist for classifying mixed pain in PMPS. Accordingly, misclassification bias cannot be excluded. Formal sensory testing and structured symptom-profiling instruments were not used, which may limit the granularity of pain characterization. Detailed medication histories were not available—although all patients had exhausted WHO analgesic ladder–based therapy prior to referral—which limits interpretation of prior treatment adequacy. Future advances in pain assessment and personalized pain profiling may help standardize diagnosis and improve clinical decision-making in mixed pain.

## Conclusion

Mixed pain in PMPS may represent a clinically meaningful pattern, characterized more by the coexistence of multiple sources than by the severity of the pain.

Clinicians treating women with persistent pain after breast cancer surgery, particularly when multiple pain sites or radiotherapy-related changes are present, should be vigilant for signs of mixed pain and employ multimodal, mechanism-based strategies.

Future research should aim to establish consensus diagnostic criteria and conduct mechanism-stratified clinical trials to facilitate more individualized and effective pain management strategies.

## Data Availability

The raw data supporting the conclusions of this article will be made available by the authors, without undue reservation.
