# Supplementary material for: Post-mastectomy pain syndrome as a model for mixed pain: clinical evidence from a specialized cancer pain clinic
Source: Front Med (Lausanne). 2026 Apr 15;13:1733623. doi: 10.3389/fmed.2026.1733623 (PMC13124505; doi:10.3389/fmed.2026.1733623)
Supplement: Supplementary file 1 [file Table_1.DOCX]

## Table 1. Clinical and Surgical Characteristics of the Study Population

| Characteristic | Mean ± SD | Number (%) |
| --- | --- | --- |
| Demographic |  |  |
| Age, years | 57.9 ± 12.5 | 120 (100%) |
|  |  |  |
| Pain Measures |  |  |
| Pain items score, total | 25.8 ± 7.8 | 90 (75.0%) |
| Pain interference items score, total | 45.0 ± 16.4 | 93 (77.5%) |
| Pain Catastrophizing Scale (PCS), total | 31.1 ± 13.4 | 81 (67.5%) |
|  |  |  |
| Pain Type |  |  |
| Nociceptive |  | 49 (40.8%) |
| Neuropathic |  | 30 (25.0%) |
| Mixed |  | 41 (34.2%) |
|  |  |  |
| Surgical Characteristics |  |  |
| Type of surgery — Plain |  | 89 (74.2%) |
| Type of surgery — With reconstructive surgery |  | 31 (25.8%) |
| Time from surgery — < 6 months |  | 18 (15.0%) |
| Time from surgery — 6–12 months |  | 32 (26.7%) |
| Time from surgery — >12 months–<5 years |  | 33 (27.5%) |
| Time from surgery — >5 years |  | 33 (27.5%) |
| Lymphedema present |  | 41 (34.2%) |

Values are presented as mean ± standard deviation (SD) or n (%). Percentages are calculated from the total cohort (N = 120). Time-from-surgery data were available for 116/120 patients (96.7%); 4 patients had missing/invalid values.

Abbreviation: PCS, Pain Catastrophizing Scale.
