# Supplementary material for: Post-mastectomy pain syndrome as a model for mixed pain: clinical evidence from a specialized cancer pain clinic
Source: Front Med (Lausanne). 2026 Apr 15;13:1733623. doi: 10.3389/fmed.2026.1733623 (PMC13124505; doi:10.3389/fmed.2026.1733623)
Supplement: Supplementary file 2 [file Table_2.DOCX]

## Appendix Table 2. Univariate Comparison of Predictors by Pain Group

Univariate analyses comparing patients with mixed pain (n = 35) to those with nociceptive or neuropathic pain (n = 85). Categorical variables analyzed using χ² tests; continuous variables using Kruskal–Wallis tests. Significant values are bolded (* p < 0.05).

| **Variable** | **Type** | **p value** | **Significant** |
| --- | --- | --- | --- |
| Pain source | Categorical | **0.0000** | * |
| Pain due to radiation | Categorical | **0.0031** | * |
| Time from surgery | Categorical | 0.3205 |  |
| PCS score | Continuous | 0.3333 |  |
| Age | Continuous | 0.3368 |  |
| Later diagnosis of fibromyalgia | Categorical | 0.5771 |  |
| Pain intervention score | Continuous | 0.5950 |  |
| Lymphedema | Categorical | 0.8418 |  |
| Systemic treatment | Categorical | 0.8701 |  |
| An interventional procedure was suggested | Categorical | 0.9057 |  |
| An interventional procedure was carried | Categorical | 0.9057 |  |
| Type of surgery | Categorical | 0.9370 |  |
| Hormonal treatment | Categorical | 0.9637 |  |
| Peripheral neuropathy | Categorical | 1.0000 |  |

Abbreviations: PCS, Pain Catastrophizing Scale. Significant differences determined at p < 0.05.

Primary pain source categories reflect the predominant anatomical and mechanistic pain generator identified during structured clinical assessment. Diffuse upper-quadrant pain and diffuse regional pain represent distinct single-mechanism entities (e.g., radicular/plexopathy or myofascial pain) and should not be interpreted as equivalent to “multiple pain sources,” which reflects the presence of ≥2 concurrent pain mechanisms identified in the same or different anatomical regions. Percentages are calculated from the total cohort (N = 120).
