# Supplementary material for: Post-mastectomy pain syndrome as a model for mixed pain: clinical evidence from a specialized cancer pain clinic
Source: Front Med (Lausanne). 2026 Apr 15;13:1733623. doi: 10.3389/fmed.2026.1733623 (PMC13124505; doi:10.3389/fmed.2026.1733623)
Supplement: Supplementary file 3 [file Table_3.docx]

**Appendix Table 3. Univariate Predictors of Mixed Pain**

Univariate logistic regression analyses comparing mixed pain versus nociceptive or neuropathic pain.

Analytic sample was restricted to complete-case data for prespecified covariates (N = 113; mixed events = 38). All 120 patients had available pain type classification; exclusions were limited to cases with missing or invalid covariate coding.

Values shown are β (coefficient), odds ratio (OR) with 95% confidence interval (CI), standard error (SE), and P value.

| Predictor | β | OR (95% CI) | SE | P value |
| --- | --- | --- | --- | --- |
| Age | -0.009 | 0.99 (0.96–1.02) | 0.016 | 0.574 |
| Type of surgery (reconstruction vs plain) | -0.291 | 0.75 (0.30–1.84) | 0.458 | 0.526 |
| Time from surgery (ordinal category) | 0.051 | 1.05 (0.73–1.52) | 0.189 | 0.787 |
| Current systemic treatment | -0.229 | 0.80 (0.26–2.45) | 0.574 | 0.690 |
| Current hormonal treatment | 0.025 | 1.03 (0.47–2.24) | 0.399 | 0.949 |
| Pain attributed to radiotherapy | 1.128 | 3.09 (0.97–9.82) | 0.590 | 0.056 |
| Multiplicity of pain sources (≥2 vs single) | 3.023 | 20.56 (7.45–56.69) | 0.518 | <0.001 |
| Lymphedema | -0.020 | 0.98 (0.43–2.23) | 0.419 | 0.962 |
| Later diagnosis of fibromyalgia | 0.533 | 1.70 (0.67–4.35) | 0.478 | 0.266 |
| Peripheral neuropathy | -0.268 | 0.76 (0.22–2.62) | 0.628 | 0.669 |
| A block was suggested | 0.304 | 1.35 (0.60–3.05) | 0.415 | 0.464 |
| A block was carried | -0.022 | 0.98 (0.44–2.17) | 0.407 | 0.957 |

Abbreviations: OR, odds ratio; CI, confidence interval; SE, standard error.
