# Supplementary material for: Post-mastectomy pain syndrome as a model for mixed pain: clinical evidence from a specialized cancer pain clinic
Source: Front Med (Lausanne). 2026 Apr 15;13:1733623. doi: 10.3389/fmed.2026.1733623 (PMC13124505; doi:10.3389/fmed.2026.1733623)
Supplement: Supplementary file 4 [file Table_4.docx]

**Appendix Table 4. Multivariable Logistic Regression: Predictors of Mixed Pain**

Logistic regression model predicting mixed pain (1 = mixed; 0 = nociceptive + neuropathic pain). Values shown are β (coefficient), standard error (SE), adjusted odds ratio (aOR) with 95% confidence interval (CI), and P value. aOR > 1 indicates higher odds of mixed pain.Analytic sample was restricted to complete-case data for prespecified covariates (N = 113; mixed events = 38).

| Predictor | Type | β (Coefficient) | SE | aOR (95% CI) | P value |
| --- | --- | --- | --- | --- | --- |
| Age | Continuous | -0.000 | 0.024 | 1.00 (0.95–1.05) | 0.997 |
| Type of surgery (reconstruction vs plain) | Categorical | -0.753 | 0.706 | 0.47 (0.12–1.88) | 0.286 |
| Time from surgery (ordinal category) | Categorical | 0.257 | 0.298 | 1.29 (0.72–2.32) | 0.387 |
| Current systemic treatment | Categorical | 0.428 | 0.814 | 1.53 (0.31–7.57) | 0.599 |
| Current hormonal treatment | Categorical | -0.242 | 0.596 | 0.79 (0.24–2.53) | 0.685 |
| Pain attributed to radiotherapy | Categorical | 1.749 | 0.822 | 5.75 (1.15–28.82) | 0.033 |
| Multiplicity of pain sources (≥2 vs single) | Categorical | 3.911 | 0.741 | 49.96 (11.69–213.41) | <0.001 |
| Lymphedema | Categorical | -0.348 | 0.660 | 0.71 (0.19–2.58) | 0.598 |
| Later diagnosis of fibromyalgia | Categorical | 1.105 | 0.703 | 3.02 (0.76–11.97) | 0.116 |
| Peripheral neuropathy | Categorical | 0.424 | 0.918 | 1.53 (0.25–9.24) | 0.644 |
| A block was suggested | Categorical | -0.549 | 0.816 | 0.58 (0.12–2.86) | 0.501 |
| A block was carried | Categorical | 0.248 | 0.785 | 1.28 (0.28–5.97) | 0.752 |

Abbreviations: aOR, odds ratio; CI, confidence interval; SE, standard error.
