# Supplementary material for: Post-mastectomy pain syndrome as a model for mixed pain: clinical evidence from a specialized cancer pain clinic
Source: Front Med (Lausanne). 2026 Apr 15;13:1733623. doi: 10.3389/fmed.2026.1733623 (PMC13124505; doi:10.3389/fmed.2026.1733623)
Supplement: Supplementary file 5 [file Table_5.DOCX]

**Supplementary Table 5. Contingency Table of Pain Source Multiplicity and Mixed Pain Classification**
Cross-tabulation of pain source multiplicity (≥2 vs single) by mixed pain classification in the analytic regression sample (N = 113; mixed events = 38; non-mixed = 75). Values shown are numbers of patients in each cell. No zero cells were observed.

| Multiplicity of Pain Sources | Non-Mixed (n = 75) | Mixed (n = 38) | Total |
| --- | --- | --- | --- |
| Single source (0) | 67 | 11 | 78 |
| ≥2 sources (1) | 8 | 27 | 35 |
| Total | 75 | 38 | 113 |
