# Supplementary material for: Post-mastectomy pain syndrome as a model for mixed pain: clinical evidence from a specialized cancer pain clinic
Source: Front Med (Lausanne). 2026 Apr 15;13:1733623. doi: 10.3389/fmed.2026.1733623 (PMC13124505; doi:10.3389/fmed.2026.1733623)
Supplement: Supplementary file 6 [file Table_6.DOCX]

**Supplementary Table 6. Firth Penalized Logistic Regression Sensitivity Analysis: Factors Independently Associated with Mixed Pain**

Sensitivity analysis using Firth’s penalized likelihood logistic regression to evaluate factors independently associated with mixed pain (mixed pain = 1; nociceptive/neuropathic pain = 0) in the complete-case analytic cohort (N = 113; mixed events = 38). Results are presented as coefficients (β), standard errors (SE), adjusted odds ratios (aOR), 95% confidence intervals (CI), and P values.

| Predictor | β | SE | Firth aOR | 95% CI | P value |
| --- | --- | --- | --- | --- | --- |
| Age | 0.010 | 0.022 | 1.01 | 0.97–1.05 | 0.653 |
| Type of surgery (reconstruction vs plain) | -0.366 | 0.600 | 0.69 | 0.21–2.25 | 0.542 |
| Time from surgery (ordinal category) | 0.187 | 0.275 | 1.21 | 0.70–2.07 | 0.496 |
| Current systemic treatment | 0.075 | 0.115 | 1.08 | 0.86–1.35 | 0.515 |
| Current hormonal treatment | 0.173 | 0.180 | 1.19 | 0.84–1.69 | 0.335 |
| Pain attributed to radiotherapy | 1.261 | 0.720 | 3.53 | 0.86–14.48 | 0.080 |
| Multiplicity of pain sources (≥2 vs single) | 3.202 | 0.633 | 24.58 | 7.11–84.95 | <0.001 |
| Lymphedema | -0.477 | 0.613 | 0.62 | 0.19–2.06 | 0.436 |
| Later diagnosis of fibromyalgia | 1.230 | 0.690 | 3.42 | 0.88–13.24 | 0.075 |
| Peripheral neuropathy | 0.400 | 0.818 | 1.49 | 0.30–7.41 | 0.625 |
| A block was suggested | -0.208 | 0.736 | 0.81 | 0.19–3.44 | 0.778 |
| A block was carried | 0.079 | 0.699 | 1.08 | 0.28–4.26 | 0.910 |

Model included the same prespecified covariates as the primary multivariable logistic regression: age, type of surgery (reconstruction vs plain), time from surgery (ordinal category), current systemic treatment, current hormonal treatment, pain attributed to radiotherapy, multiplicity of pain sources (≥2 vs single), lymphedema, later diagnosis of fibromyalgia, peripheral neuropathy, and interventional block variables (block suggested and block carried). Firth penalized estimation was used to reduce small-sample bias and mitigate potential quasi-separation. The analytic sample was restricted to complete-case covariate data.

**Abbreviations**

**aOR**, adjusted odds ratio; **CI**, confidence interval; **SE**, standard error.
